# Simultaneous electrochemical determination of morphine and methadone by using CMK-5 mesoporous carbon and multivariate calibration

**DOI:** 10.1038/s41598-022-12506-9

**Published:** 2022-05-18

**Authors:** Mohammad Mehdi Habibi, Jahan B. Ghasemi, Alireza Badiei, Parviz Norouzi

**Affiliations:** 1 School of Chemistry, College of Science, University of Tehran, Tehran, Iran; 2 Center of Excellence in Electrochemistry, Department of Chemistry, University of Tehran, Tehran, Iran

**Keywords:** Chemistry, Analytical chemistry, Bioanalytical chemistry

## Abstract

For the first time, a sensitive electrochemical sensor using a glassy carbon electrode modified with CMK-5 Ordered mesoporous carbon was fabricated for simultaneous analysis of morphine and methadone. Modern electrochemical FFT-SWV techniques and partial least-squares as a multivariable analysis were used in this method. CMK-5 nanostructures were characterized by field emission scanning electron microscopy, transmission electron microscopy, X-ray diffraction analysis, and Raman spectroscopy. Variables such as accumulation time and pH for the proposed sensor were optimized before quantitative analysis. To train the proposed sensor, standard mixtures of morphine (MOR), and methadone (MET) were prepared in the established linear ranges of the analyzes. The results obtained from training samples were used for PLS modeling. The efficiency of the model was determined using test and real matrix samples. The root mean square error of prediction and the squared correlation coefficients (R^2^_p_) for MET and MOR were estimated to be 0.00772 and 0.00892 and 0.948 to 0.990, respectively. The recoveries in urine samples were reported to be 97.0 and 105.6% for both MOR and MET, respectively.

## Introduction

Morphine (MOR) is an analgesic of the opiate family commonly used to relieve acute pain. However, it can become habit-forming and lead to physical dependence^1^. It is also classified as a banned doping agent for athletes^2^. Methadone (MET), also known as 6-dimethylamino-4, 4-diphenyl-3-heptanone, is a potent synthetic analgesic used as an opioid for maintenance therapy in the treatment of heroin and morphine addicts and for the treatment of chronic pain management^3,4^. However, research has also shown that MET can be addictive, and addiction to MET is increasing in various societies^5,6^. According to the study results, MOR and MET have almost similar pharmacological properties, and overdose of MOR and MET can lead to premature death in people^7^.

In the last decades, researchers have developed a variety of methods for the analysis of MOR and MET, such as high-performance liquid chromatography, supercritical fluid chromatography, gas chromatography coupled with mass spectroscopy, chemiluminescence, spectrophotometry, which are generally time-consuming, prohibitively expensive, complicated, and often require pretreatment^8–14^. Electrochemical techniques are emerging as promising alternative methods for pharmaceutical analysis because of their rapid results, simple operation, accuracy, and cost-effectiveness^15^. Furthermore, the fast fourier transform square wave voltammetry (FFT-SWV) technique has been used as a sensitive and accurate method for the analysis of various compounds. FFT-SWV uses Fourier transform filtering combined with electrochemical methods to eliminate instruments' stationary noises, which helps to improve sensitivity^16,17^.

However, electrochemical detection of MOR and MET with bare electrodes has several limitations, such as slow electron transfer, low sensitivity, and impurities. The development of nanotechnology has provided a variety of nanomaterials as electrode surface modifiers to improve the electrochemical detection of MOR and MET. Carbon nanostructures such as carbon nanotubes and graphene have been widely used to fabricate electrochemical sensor for MOR and MET^18–20^. For example, Behzad Rezaei et al.^21^ have developed an electrochemical sensor based on carbon nanotubes and carbon quantum dots to detect methadone. Also, Maccaferri et al. have used exfoliated graphene oxide to fabricate an electrochemical detection of morphine^22^. The results show that carbon nanotubes and graphene can improve the electroanalytical performance of the sensor due to their large surface area, electrical conductivity, and electrocatalytical properties^23^. CMK-5 is a group of ordered mesoporous carbon with high porosity and a large surface area. Recent research has shown that ordered mesoporous carbon has lower electron transfer resistance than carbon nanotubes^24^. Due to the extremely well-ordered pore structure, the high specific pore volume, the high specific surface area and the good electrochemical conductivity, OMCs are suitable for use in fields of electrochemical sensor technology^25^. For example, OMCs have been used electrochemical detection of glutathione, dopamine, glucose, and morphine^26–28^. Moreover, the high ability of these OMCs to adsorb and accumulate analytes can lead to very sensitive electrochemical sensors^29^.

**Figure 1:**
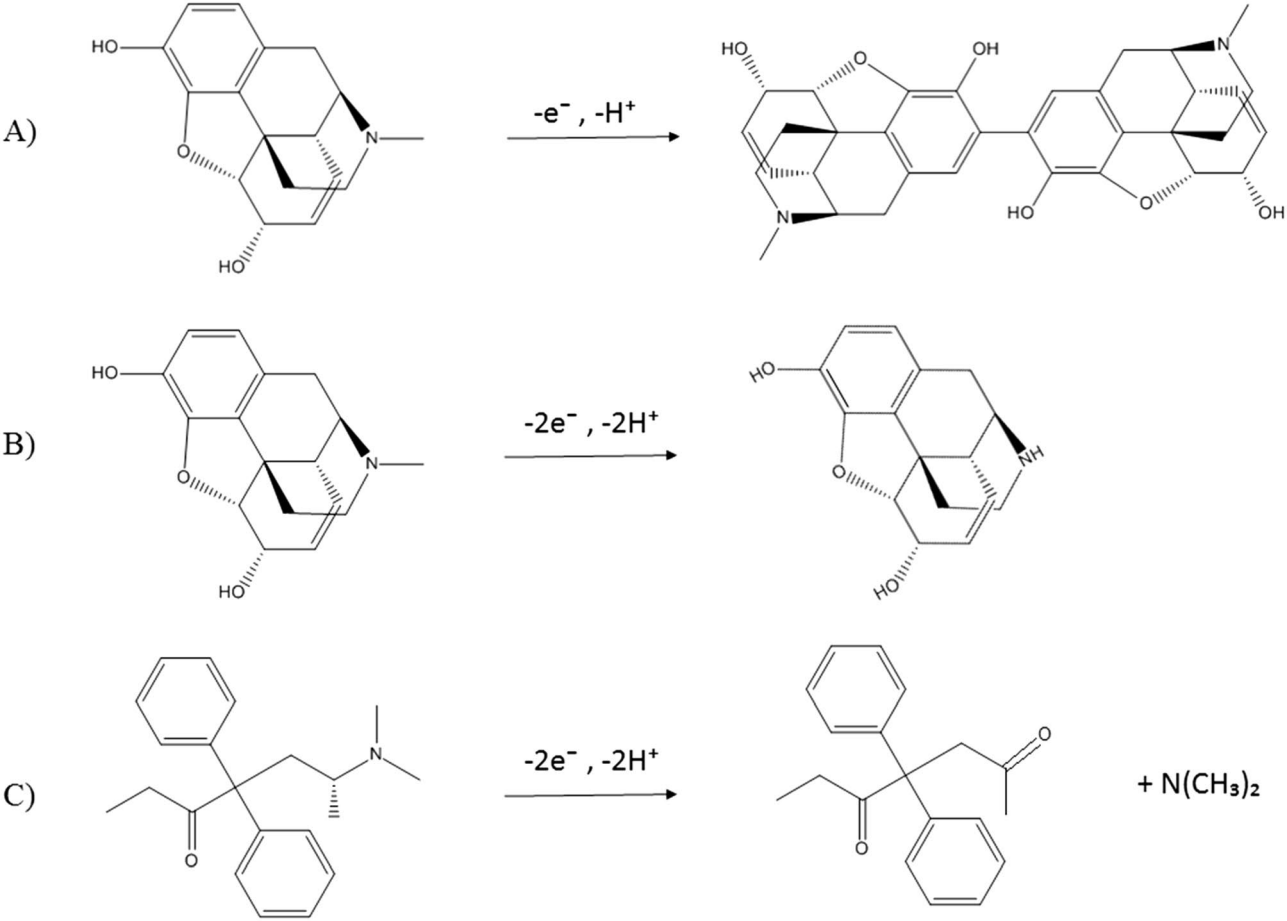
Electrochemical reaction of morphine and methadone.

Based on previous studies, the oxidation mechanism of MOR and MET substances can be written as Fig. 1

Figure 1A,B show two oxidation reactions for MOR: Fig. 1A involves the mechanism of MOR dimerization and conversion to pseudomorphine with the exchange of one electron and one proton. Figure 1B consists of the conversion of MOR to neromorphine with the exchange of two electrons and two protons. Therefore, MOR exhibits two distinct electrochemical peaks^30–32^. MET is oxidized by the mechanism shown in Fig. 1C, in which one electron and one proton are exchanged. Therefore, it shows only one electrochemical peak^33^. A review of previous articles shows that the second peak of MOR, appears at a higher potential and overlaps with the electrochemical peak of MET^34^.

Multivariate calibrations can be used to solve the problem of signal overlap. Unlike univariate calibration methods, which evaluate the signal of one analyte, the responses of several combinations are considered simultaneously^35–37^. Compared to other types of multivariate calibrations, the partial least-squares (PLS) technique has many advantages as a linear model has many advantages, such as providing high performance, use of all response profiles, reduction of interference effects, and ignoring concentrations of components other than the desired analyte^38–41^.

**Figure 2:**
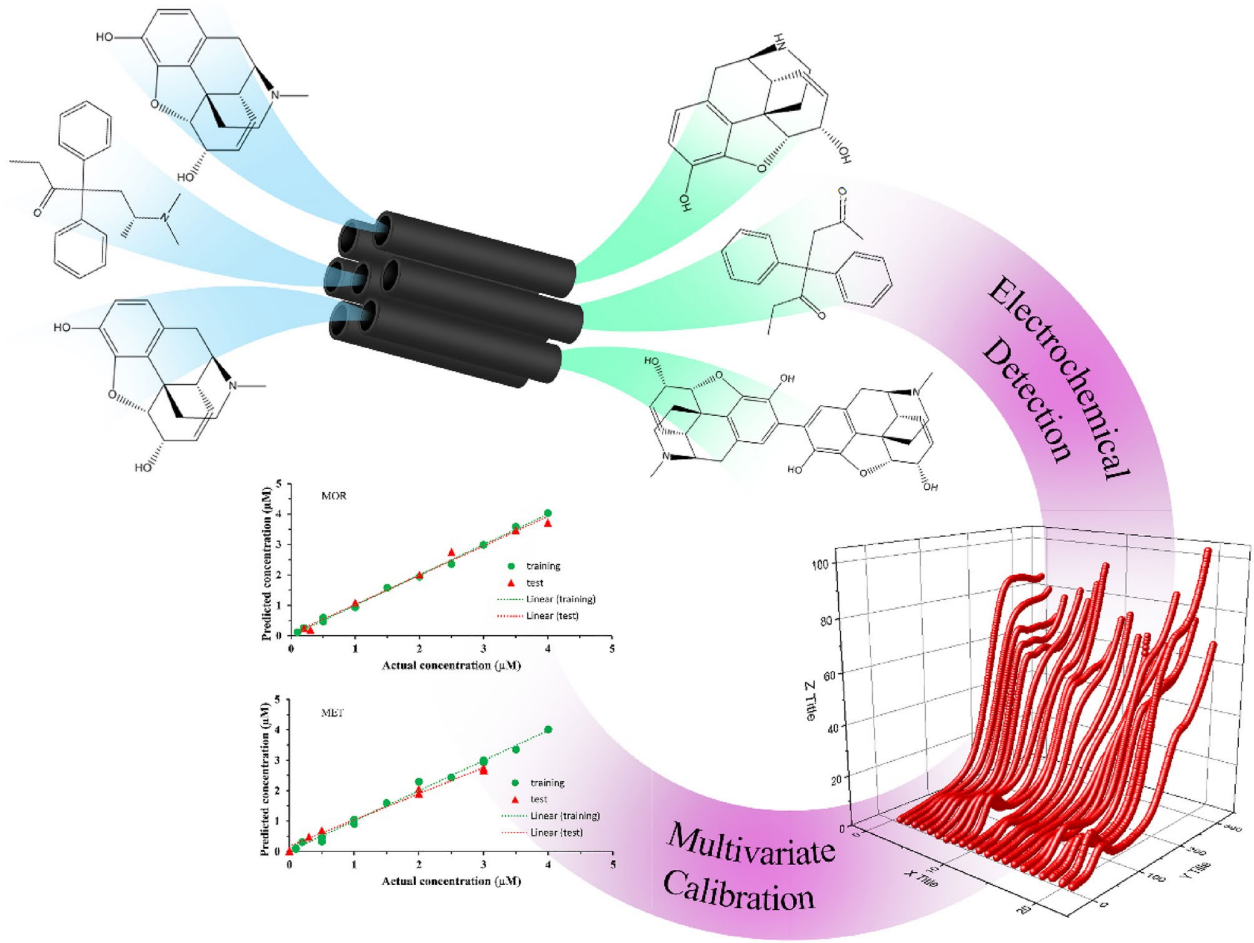
Schematic representation of the simultaneous electrochemical detection of MOR and MET at CMK-5 Mesoporous Carbon Surface by a combination of FFT-SWV with Multivariate Calibration.

This work focuses on the synthesis of CMK-5 mesoporous carbon nanostructures to modify the surface of a glassy carbon electrode (GCE/CMK-5) for simultaneous measurement of MOR and MET. CMK-5 was characterized by X-ray diffraction analysis (XRD), Raman spectroscopy, scanning electron microscopy (SEM), and transmission electron microscopy (TEM). In addition, the electrochemical properties of the modified GCE/CMK -5 electrode were investigated by cyclic voltammetry (CV) and electrochemical impedance spectroscopy (EIS). The FFT-SWV technique was used for quantitative analysis. The digitized response profiles were used as input for the multivariate calibration can be observed in Fig. 2. Finally, the applicability of the sensor in measuring MOR and MET in urine was investigated as a real sample.

## Material and methods

### Materials

pluronic P123, tetraethyl orthosilicate (TEOS), aluminium chloride, furfuryl alcohol (FA), trimethylbenzene (TMB), oxalic acid (OA), N, N-dimethylformamide (DMF) were purchased from Sigma-Aldrich. All other reagents were of analytical grade with maximum purity. Morphine (MOR) and methadone (MET) were supplied by the Darou Pakhsh Holding Company (DPHC). All the solutions were prepared using high purity water from a Millipore system (> 15 M cm). Phosphate buffer solution (PBS, 0.1 M, pH 8.5) was employed as a supporting electrolyte. The stock solutions of MOR and MET were made separately at concentrations of 1 mM in PBS and stored in the refrigerator. Also, the working solutions used for analyses were obtained by diluting the stock solution with PBS.

### Synthesis of CMK-5

**Figure 3:**
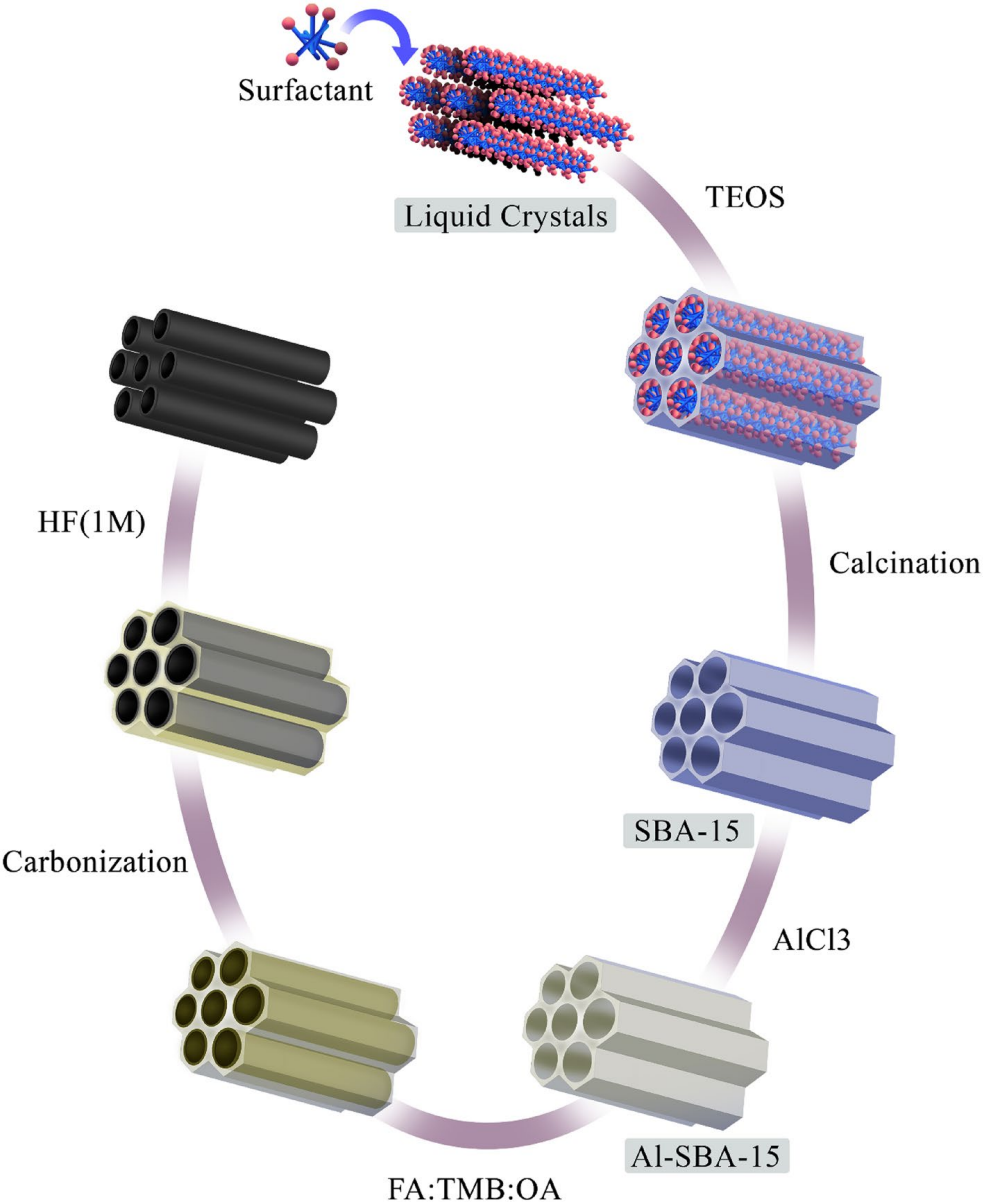
Schematic representation of the preparation procedure of CMK-5 mesoporous carbon by the nanocasting strategy using an Al-SBA-15 hard template.

The synthesis of CMK-5 mesoporous carbon is shown in Fig. 3. In the first step, SBA-15 was synthesized according to the method described by Zhao et al.^42^. briefly a liquid crystal template was prepared by dissolving 4 g of P123 surfactant in 150 mL of HCl (2 M) aqueous solution. Then 9.6 mL TEOS was added drowsily to the above solution under stirring at 40 °C stirring for 20 h and then heated in an autoclave at 100 °C for 24 h. The obtained solid product was separated, washed, and dried at 60 °C. Finally, the dried powder was calcined at 550 °C for 8 h to remove the template. For the synthesis of Al-SBA-15, 2.0 g of SBA-15 was ultrasonically dispersed in an ethanolic solution of 16.6 mM AlCl_3_ for 2 h. The ethanol was then evaporated in a vacuum oven and the remaining solid was calcined at 550 °C for 3 h^43^. CMK-5 was synthesized in a template of Al-SBA-15. To prepare CMK-5, a solution mixture of furfuryl alcohol (FA) and trimethylbenzene (TMB) as carbon sources and oxalic acid (OA) as catalyst was prepared at a molar ratio of 200:185:1. This solution was mixed with Al-SBA-15 and stirred at a temperature of 80 °C for 1 h. The suspension was dried in an air oven at 150 °C for 12 h. Consequently, aluminum silicate and oxalic acid catalyzed the polymerization of furfuryl alcohol on the pore wall of Al-SBA 15.The obtained dark brown powder was carbonized in a furnace at 300 °C with a heating rate of 1 °C min^−1^ and then 800 °C with a rate of 5 °C min^−1^ for 4 h under nitrogen atmosphere. Finally, the product was treated with 20 mL of HF (1 M) aqueous solution to remove the silicate template.

### Preparation of CMK-5 mesoporous carbon-modified GCE

GCE/CMK-5 was prepared as follows: First, the GCE (2 mm) was polished to achieve a mirror-like surface with 0.3 and 0.05 μm alumina. Then, the electrode was sonicated in a mixture of water and ethanol (50:50) for 5 min to clean the electrode surface and finally dried under high purity nitrogen flow. Next, 1 g of CMK-5 was weighed and dispersed in 1 mL of DMF by sonication for 15 min. Eventually, about 2 µL of the prepared suspension was dropped onto the surface of the GC electrode and dried under the IR lamp. The GCE/CMK-5 electrode was obtained.

### Apparatus and measurements

X-ray diffraction analysis (XRD) was performed on a Philips diffractometer of X'pert company device with monochromatic Cu Kα radiation (λ = 1.5406 Å). The Raman spectroscopy was recorded by a device of Takram N1-541 model (laser wavelength 532 nm) produced by Teksan Company. Transmission electron microscopy (TEM) image was obtained by Philips CM30 with an accelerating voltage of 200 kV. Scanning electron microscopy (SEM) images were obtained by TeScan–Mira III as well as energy-dispersive X-ray spectroscopy (EDS, Tescan, VEGA-3 LMU VPSEM, Czech Republic). The pH meter (Metrohm 744 pH Meter) was used. The sonication bath (B8510, Branson Ultrasonic Corporation) was used for ultrasonic radiation.

### Electrochemical tests

**Figure 4:**
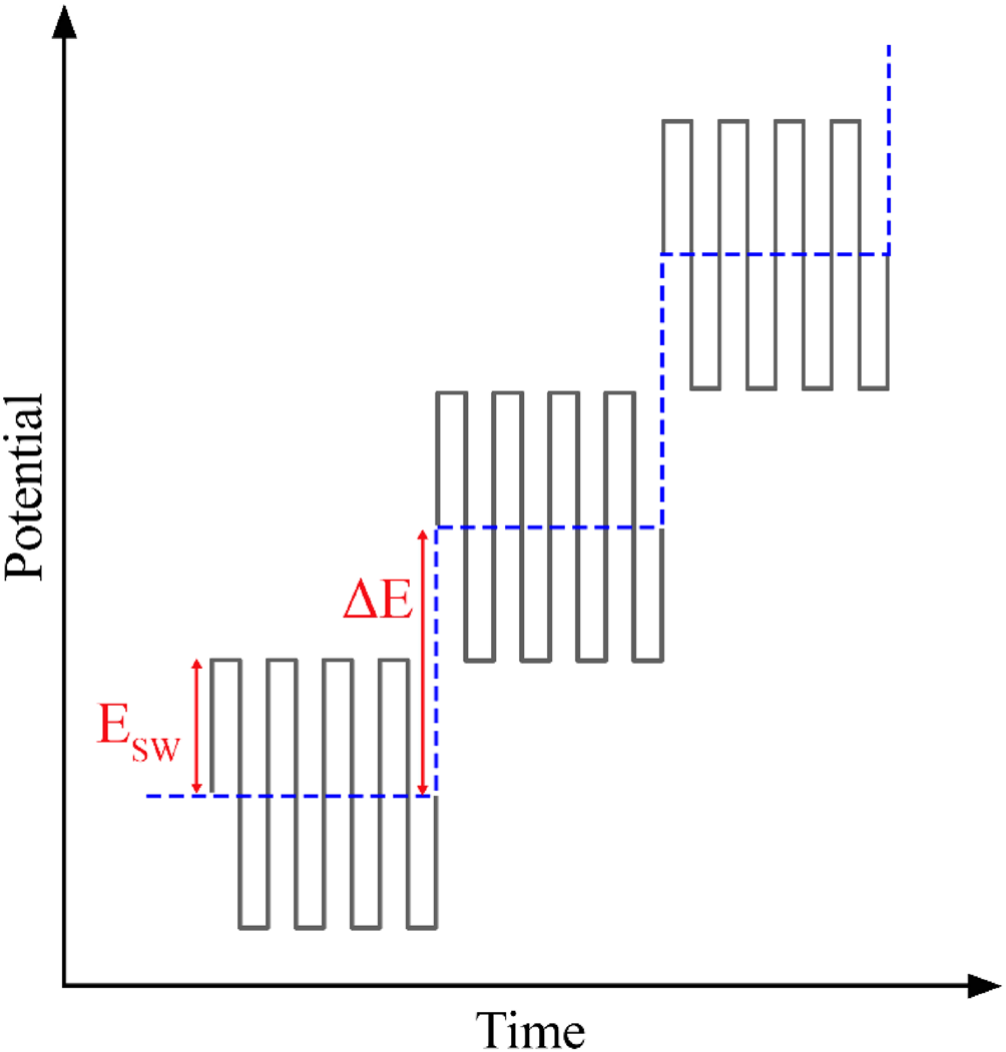
Applied potential waveform during FFT-SWV measurements.

All electrochemical experiments were performed by μStat-i 400 s as a portable Potentiostat/Galvanostat/Impedance Analyzer (Metrohm Drapsens, The Netherlands) controlled by a personal computer with DropView 8400 software^44^. It has a three-electrode system, including a GCE (2 mm in diameter) as the working electrode, a platinum wire as the auxiliary electrode, and an Ag/AgCl electrode as the reference electrode. fast fourier transform square wave voltammetry (FFT-SWV) is a modified technique based on the square wave voltammetry (SWV) technique using the discrete fast fourier transform (FFT) method for background subtraction and two-dimensional integration of the electrode response over a selected potential range and time window. As shown in Fig. 4, multiple square wave (SW) pulses of defined amplitude and frequency were superimposed on a staircase potential function and currents were sampled 4 times per SW polarization cycle. This technique is performed by a homemade potentiostat controlled by a computer equipped with an analog–digital board. In this study, FFT-SWV measurements were performed by applying a potential scan from 0 to 1.1 V at a frequency of 256 Hz and the amplitude of 15 mV.

### Multivariate calibration

Partial least squares (PLS) were employed to construct a predictive model based on the voltammetric profiles for different MOR and MET concentrations. Solutions of binary mixtures of MOR and MET ranging from 0.1 to 4 µM were prepared, including 14 training samples and 6 test samples.

Briefly, PLS consists of an input matrix X and an output matrix Y, following the general formula.

1 $$ Y = XC + Y $$

C and V are coefficient and noise matrices in this formula, respectively. The general model of PLS regression is built from a bilinear model by decomposing matrices X and Y as follows:

2 $$ X = t_{l} p_{l}^{T} + E_{l} $$

3 $$ Y = u_{l} q_{l}^{T} + F_{l} $$

where t_1_ and u_1_ are latent score vectors of the first PLS factor, p_1_ and q_1_ are corresponding loading vectors, and E_1_, F_1_ are error matrices^45,46^.

Since the number of latent variables should be neither too high nor too low, as this leads to overfitting or underfitting when modeling the data, the evaluation of the multivariate model by statistical parameters to determine the correct number of LVs is of high importance. The statistical parameters include squared correlation coefficient (R^2^), root mean square error of calibration (RMSEC), root mean square error of prediction (RMSEP), and root mean square error of cross-validation (RMSECV). The R^2^ value was calculated as

4 $$ R^{2} = \mathop \sum \limits_{i = 1}^{n} \left( {\hat{y} - \overline{y}} \right)^{2} /\mathop \sum \limits_{i = 1}^{n} \left( {y_{i} - \overline{y}} \right)^{2} $$

where *y*_i_ is the actual concentration of the analyte in sample *i*, $\hat{y}_{i}$ represents the estimated concentration of the analyte in sample *i*, $\overline{y}$ is the mean of the actual concentration in the calibration set, and *n* is the total number of samples used in the calibration set. The RMSEC, RMSEP and RMSECV were calculated as:

5 $$ RMSEC = \left[ {\frac{1}{N}\mathop \sum \limits_{i = 1}^{n} \left( {y_{i} - \hat{y}_{i} } \right)^{2} } \right]^{1/2} $$

6 $$ RMSEP = \left[ {\frac{1}{p}\mathop \sum \limits_{i = 1}^{p} \left( {y_{i} - \hat{y}_{i} } \right)^{2} } \right]^{1/2} $$

7 $$ RMSECV = \left[ {\frac{1}{N}\mathop \sum \limits_{i = 1}^{n} \left( {y_{i} - \hat{y}_{cv} } \right)^{2} } \right]^{1/2} $$

### Ethical declaration

The whole procedure of this study is per the ethical standards of the institutional and national research committees and with the 1964 Helsinki declaration and its later amendments or comparable ethical standards. Informed consent was obtained from all the subjects and/or their legal guardians. And this process was carried out under the supervision of the Ethics Committee of the University of Tehran.

## Results and discussion

### Characterization

The powder X-ray diffraction (XRD) patterns of Al SBA-15 and CMK-5 are shown in Fig. S1A. The diffraction pattern of Al-SBA-15 shows a sharp peak at 0.92° corresponding to reflections (100) arrays and two weak peaks at 1.78° and 2.02° assigned to the (110) and (200) planes, indicating a 2D hexagonal microstructure with a p6mm space group. Also, the presence of the reflections of (100), (110), and (200) in the XRD pattern of CMK-5 shows a good similarity with Al SBA-15. A shift in the position of peaks toward high angles and a decrease in the intensity of (110) and (200) peaks can be due to the smaller d-spacing and negligible shrinkage of the cell parameters in the unit cell and the structural shrinkage of CMK-5. In addition, the Raman spectrum in Fig. S1B shows the two peaks at 1340 and 1590 cm^−1^, assigned to disordered graphite (D band) and crystalline graphite (G-band), respectively.

**Figure 5:**
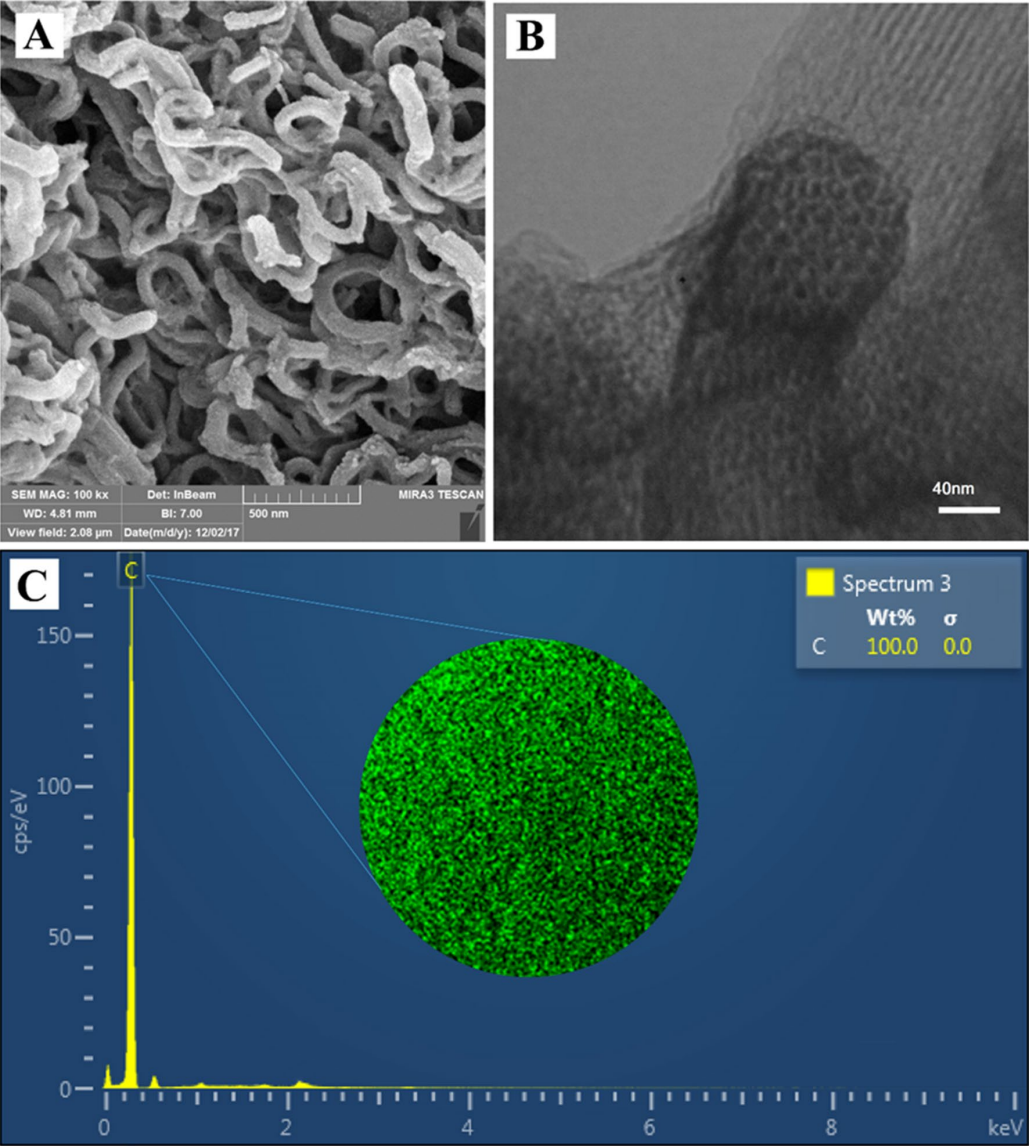
(**A**) SEM, (**B**) TEM, (**C**) EDS spectrum and EDS mapping image of CMK-5.

The morphological properties of CMK-5 were studied via SEM and TEM images. Figure 5 A shows the SEM image of twisted mesoporous carbon pipes CMK-5 with an almost smooth surface. Furthermore, mesochannels morphologies of synthesized CMK-5 can be seen in the TEM image (Fig. 5B). This image also confirms the hexagonal structure inferred from the XRD. The EDS spectrum of CMK-5 confirms the presence of carbon element and clearly shows that the AlSBA-15 hard template has been completely removed. EDS mapping illustrates the distribution of C element on the CMK-5 surface (Fig. 5C).

**Figure 6:**
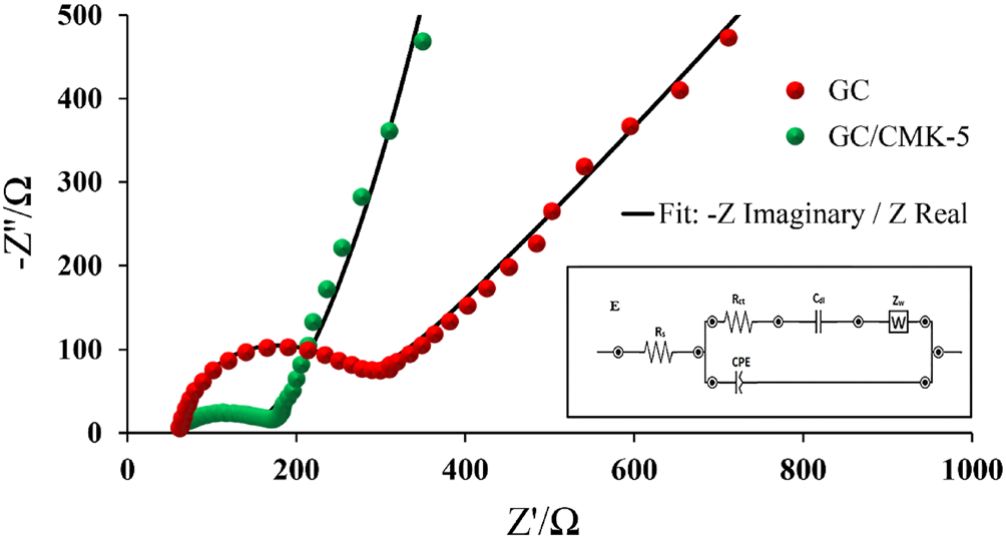
EIS characterization of GC and GC-CMK-5 electrode in 5.0 mM Fe(CN)_6_^−3/4^ containing 0.1 M KCl with the frequency range of 0.1 Hz to 100 kHz at an amplitude of 10 mV.

The electrochemical feature of CMK-5 surface was evaluated by electrochemical impedance spectroscopy. Figure 6 shows the Nyquist plot of the GCE and GCE -CMK-5. It was found that the solution resistance is almost constant. The semicircle shape of the Nyquist plot (high frequency) corresponds to the charge transfer reaction between the electroactive species in the solution and the electrode surface. The smaller the semicircle radius, the lower the resistance of charge transfer. The fitting parameters of the equivalent circuit for charge transfer (R_ct_) for GCE and GCE/CMK-5 are 189.28 and 88 Ω and C_dl_ for GCE and GCE/CMK-5 are 36.3 and 113.2 µF, respectively. The obtained the equivalent circuit values indicate CMK-5 can provide an excellent electron passage at the electrolyte/electrode interface and a high surface area for absorption of MOR and MET.

**Figure 7:**
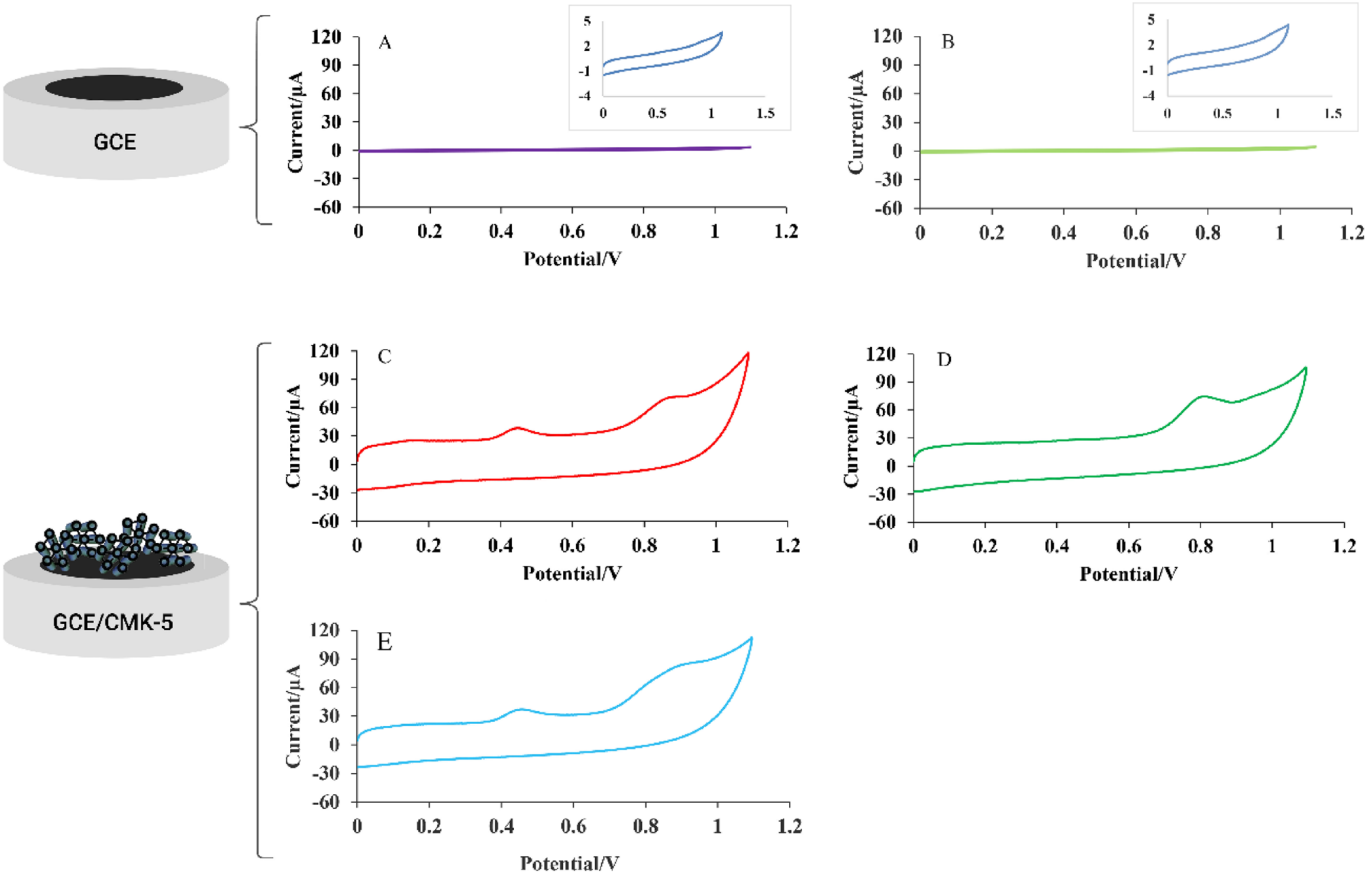
(**A**) and (**B**) Cyclic voltammograms of GC electrode presence of individual MOR, MET. (**C**) and (**D**) GC/CMK-5 electrode presence of individual MOR, MET and (**E**) GC/CMK-5 electrode presence of MOR, MET mixture.

Cyclic voltammetry was applied to investigate the electrochemical behavior of MOR and MET on the GC and GC-CMK5 electrode's surface. Figure 7 shows the cyclic voltammograms of GCE and GCE/CMK5 in 0.1 M phosphate buffer (PB) solution (pH = 8.5 ) with a scan rate of 0.1 V/s in the potential range of 0 to 1.1 V in the presence of MOR, MET with a concentration of 2 µM. Figure 7A,B, respectively, showed MOR and MET cyclic voltammograms on the GC electrode. It does not show significant anodic and cathodic peaks. In contrast, the GC-CMK-5 modified electrode (Fig. 7C,D)showed two anodic peaks at a potential of 0.45 and 0.9 V for MOR and an anodic peak for methadone at a potential of 0.81 V. Amplification of the current and decreasing of the oxidation potential of MOR and MET in the presence of CMK-5 indicate the excellent electrocatalytic properties of CMK-5. These properties are probably due to the larger surface area and the many edge plane defect sites on the surface of CMK-5, which enhances the electron transfer at the surface. In addition, the simultaneous presence of morphine and methadone disrupts the faradic currents (Fig. 7E).

### Optimization of pH and accumulation time

**Figure 8:**
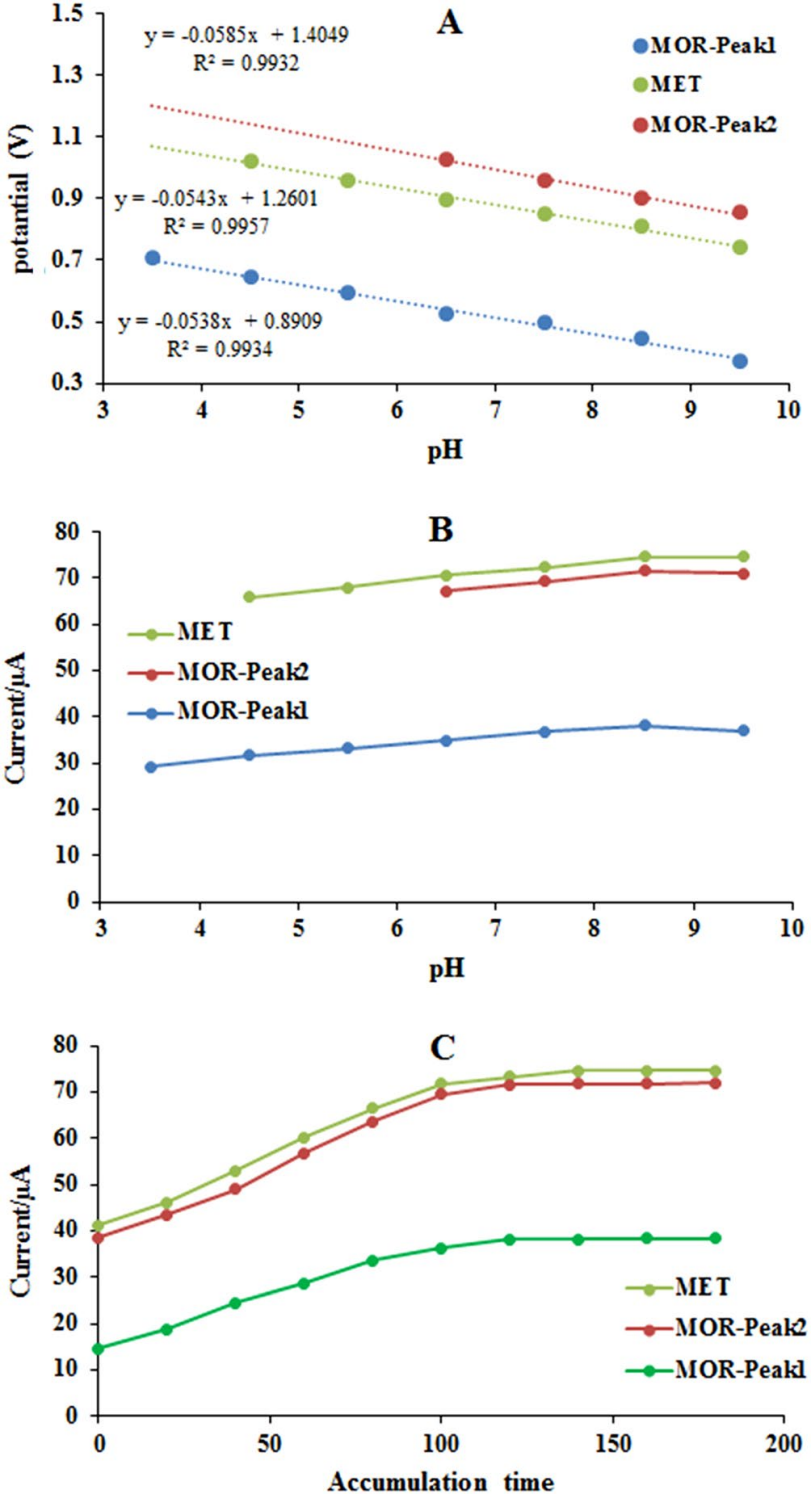
(**A**) Calibration plot of pH versus potential and (**B**) optimization plot of pH versus current. (**C**) Optimization of accumulation time (0–180 s) of 2 µM of MOR and MET in PBS. The FFT-SWV frequency of 256 Hz and the amplitude of 15 mV.

The effect of buffer pH as an important variable on the response of the electrode in the simultaneous determination of MOR and MET was evaluated. As shown in Fig. 8A, when pH was increased from 3.5 to 9.5 anodic peak 1 of MOR shifted to negative potentials with a linear trend of dependence between current and pH of 0.0538. also, incrising pH from 6.5 to 9.5, anodic peak 2 of MOR shifted to negative potentials with a line slope of 0.0585. In the case of MET, as pH increased from 4.5 to 9.5, the anodic peaks shifted to more negative potentials, and current was linearly dependent on pH with a slope of 0.0543. The linear correlation between variations in potentials and pH for the anodic peaks of morphine and methadone with a slope of 0.059, indicates an equivalent ratio of electrons and protons consumed in the electrochemical reaction^47^. However, Fig. 8B indicates both MOR anodic peaks, that the current increased by increasing pH to 8.5 and after decreased when pH increased to 9.5. Furthermore, MET anodic peak current was raised with the changing pH from 4.5 to 8.5 and then reached a constant value in the pH range of 8.5–9.5. Therefore, the supporting buffer solution pH 8.5 was selected as the optimal value.

One of the variables that can improve the electrochemical signal of MOR and MET on the electrode modified with CMK-5 is the accumulation time due to the strong adsorption properties of CMK-5. Here, the effect of accumulation time for MOR and MET from 0 to 180 s was separately investigated. The results showed (Fig. 8C) that both MOR anodic peaks grew with increasing accumulation time to 120 s. And after 120 s, they leveled off. The MET electrochemical signal increased with increasing accumulation time up to 140 s and remained constant. Finally, because the electrochemical signal for both MOR and MET was leveled off after 140 s of accumulation time, the 140 s were selected as the optimal accumulation time.

### Simultaneous measurement of MOR and MET

**Figure 9:**
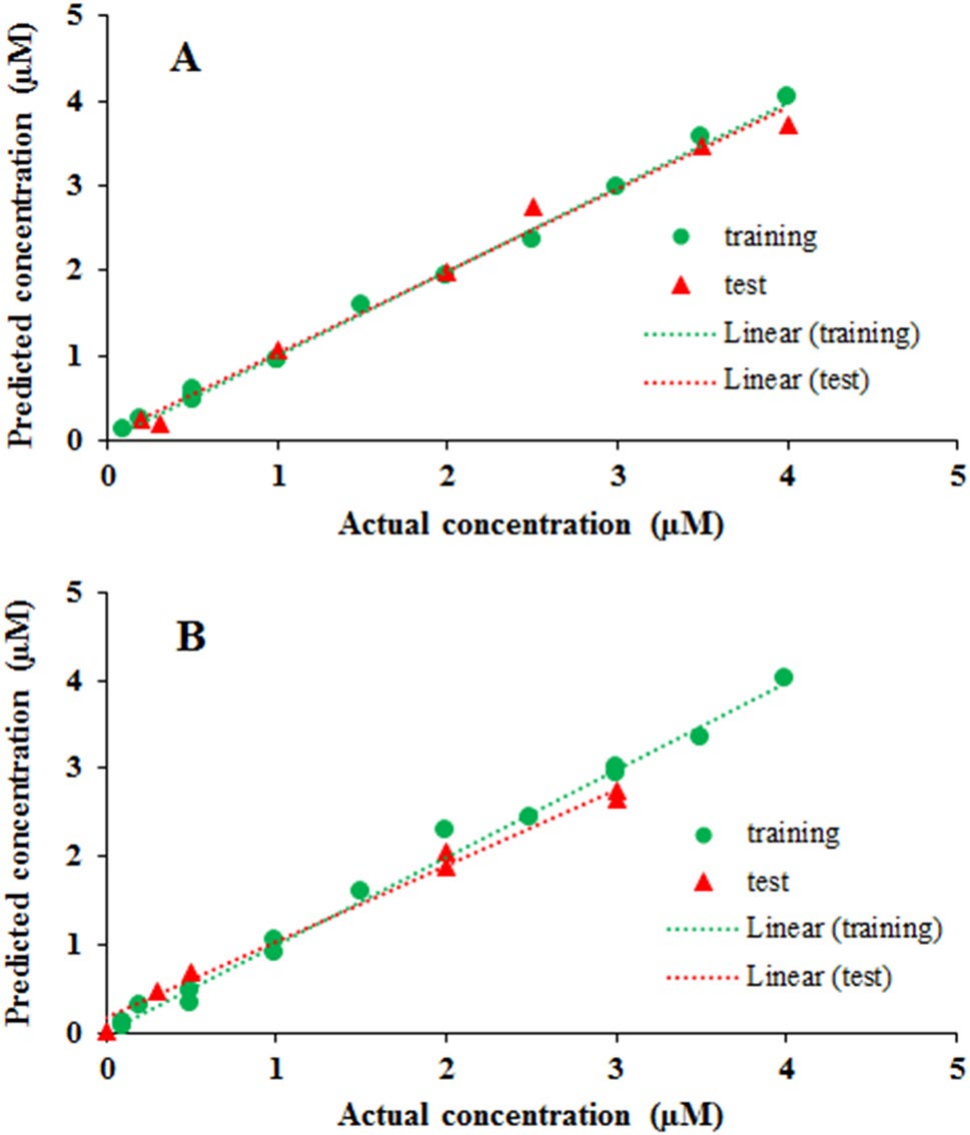
Results of Calibration and validation by PLS model for (**A**) MOR and (**B**) MET.

To obtain the linear calibration range of current as a function of concentration for both analytes, their electrochemical behavior was first studied in a univariate manner using the FFT-SWV technique. The linear equations obtained are shown in Table 1. Partial least squares (PLS) was used to construct a predictive model between the voltammetric profiles for different concentrations of MOR and MET. According to the results in Table 1, the concentrations of the solutions MOR and MET were randomly changed in the range of 0.1–4 µM for the generation of the training and test calibration matrices. The data were divided into a training set of 14 samples and a test set of 7 samples and concentrations and are shown in Table S1. Voltammograms of these mixtures were recorded according to the FFT-SWV technique and are shown in Fig. S2. The results of measuring MOR and MET in training and test samples using PLS are shown in Fig. 9A,B, respectively. They show reasonable agreement between the actual concentrations and the predicted concentrations for MOR and MET. In general, the performance of the model can be evaluated with statistical parameters, including RMSECV, RMSEP, R^2^_c_ and R^2^_p_. The most efficient model was selected based on the lowest RMSECV and RMSEP and the highest R^2^c and R^2^p. The efficiency of the model was evaluated for different latent variables (LV), and LV4 was chosen as an optimal condition. All statistical parameters for the final model are can be found in Table 3. As can be seen in Table 2, the performance of the model for MOR is better, possibly due to the fact that MOR is modeled by two parts of the voltammogram data. The part around the first peak at a potential of 0.45 responds specifically to MOR.

**Table 1:** Regression equations, calibration coefficient, and detection limit of GC/CMK-5 electrode for individual detection of MOR and MET.

Analyt	Calibration equation	R^2^	LOD (µM)
MOR Peak (1)	y = 2.434x + 4.931	0.998	0.049
MOR Peak (2)	y = 11.939x + 6.6953	0.9988	0.027
MET	y = 15.441x + 0.3945	0.999	0.021

**Table 2:** Evaluation of PLS results for the detection of MOR and MET.

Analyt	LVs	RMSEC	RMSEP	$R_{c}^{2}$	$R_{p}^{2}$
MOR	4	0.00642	0.00772	0.9973	0.9946
MET	4	0.00855	0.00892	0.987903	0.982807

Furthermore, a comparison of the present method with methods described in the literature is presented in Table 3. A thorough literature search revealed that the proposed method for multivariate electrochemical simultaneous determination of MOR and MET has no similar record.As shown in Table 3, most studies were conducted to measure MOR or MET, and simultaneous measurement of these two drugs was rarely performed. In addition, some of the previously reported methods analyzed morphine and methadone simultaneously using a univariate calibration method in which each drug was calibrated while the other drug acted as an interference. Because the separation of the peaks in these methods is not ideal and the mass transfer of the drugs can affect each other, this limits the application of the method. However, with the multivariate calibration method, morphine and methadone can be calibrated simultaneously. Moreover, the performance of the CMK-5 combined with the multivariate calibration and the FFT-SWV technique is superior to previous methods. The advantages related to the electrocatalytic properties of CMK-5 and the ability of the PLS method to correct the modeling and eliminate the most noisy part of the recorded electrochemical responses. In addition, the FFT-SWV technique effectively eliminates noise (instrument noise, thermal noise, etc.) at low concentrations using FFT filters, thereby increasing the signal-to-noise ratio and improving sensitivity. It is noteworthy that the method used in this study is simple and has good sensitivity, reproducibility, and linear range for simultaneous determination of morphine and methadone.

**Table 3:** Comparison of the proposed sensor for MOR and MET with reported detection methods.

Detection method	Calibration	Detection limit		Linear range		References
		MOR	MET	MOR	MET	
Gold/graphene screen-printed electrode		0.09	–	0.1–100	–	^48^
NiO/ MWCNT paste electrode		0.14	–	0.34–12	–	^49^
Mesoporous carbon/glassy carbon electrode		0.01	–	0.1–20	–	^30^
MWCPE		–	0.3	–	0.5–300.0	^50^
Gr/AgNPs/GCE		–	0.12	–	1.0–200.0	^18^
CQD-MWCNT nanocomposite		–	0.03	–	0.1–225	^21^
DPV(β-MnO2 nanoflowers)	Univariate	0.0083	0.0056	0.1–250	0.1–200	^33^
SWV(MMCPE)	Univariate	0.0016	0.003	0.005–1.8	0.01–8	^34^
FFT-SWV (GCE/CMK-5)	PLS	0.027	0.029	0.1–4	0.1–4	

### Interference study

To investigate the effect of interference on the simultaneous measurement of MOR and MET, common interfering species in the urine matrix were evaluated by spiking solutions with 0.5 µM MOR and MET under optimal conditions. A maximum amount of 5% of the relative prediction error for each species was determined as the tolerance limit. However, the results showed that none of the common interferences significantly affected the PLS prediction. The data obtained are shown in Table S2. As can be seen from the data in Table S2, the sensor is highly selective for the determination of MOR and MET in the presence of interfering substances.

### Reusability of the electrochemical sensor

The reusability of the GCE/CMK-5 sensor was investigated with 10 times measurement by FFT-SWV in a mixture of 0.5 µM MOR and 0.5 µM MET in PBS solution (pH = 8.5). The sensor was washed with deionized water after each measurement. The relative PLS prediction error was studied as a reusability parameter. Figure S3 shows the FFT-SWV voltammograms 1–10 of the mixture of morphine and methadone. The relative PLS prediction error of morphine and methadone after 10 tests was 3.3% and 5.7%, respectively, indicating good reusability of the sensor for measuring morphine and methadone.

### Determination of MOR and MET in Urine

Samples were prepared as follows. First, 3 urine samples were collected from healthy volunteers. Then, a certain amount of each of the drugs MOR and MET was added. To induce protein precipitation and protonation, 20 μl of a 5 M HCl solution was added to the samples. The sample was then filtered using a 0.2 um filter. To reduce interference and control pH, samples were diluted 5 times with phosphate buffer solution (pH = 8.5). Figure S4 shows FFT-SWV voltammograms of MOR (0.5 µM) and MET (0.5 µM) in pretreated urine samples on GCE/CMK-5. In this voltammogram, the oxidation peak of MOR and MET was observed. There is no significant interference with the measurement of morphine and methadone with GCE/CMK-5 in the urine sample. The applicability of the proposed electrochemical sensor was investigated in the direct measurement of MOR and MET in real matrix urine samples treated with the method described above. The final concentrations obtained are listed in Table S3. Each sample was measured 5 times, and the RSD and recovery values ranged from 2.4 to 3.9 and 95.6 to 105.6, respectively (Table S3).

## Conclusions

In conclusion, CMK-5 was successfully synthesized in a hard templated Al SBA-15 by chemical and thermal treatment methods. Investigations showed the electrocatalytic effect of CMK-5 double-pore systems on the oxidation of MOR and MET. Also, electrochemical and structural characteristics showed that CMK-5 has a high ability to absorb both drugs in its structure. The result of FFT-SWV indicated the good sensitivity of GCE/CMK-5 the detection of MOR and MET. Furthermore, Combining FFT-SWV with the partial least squares (PLS) method was used to decrease interference MOR and MET. the model obtained by this method successfully detected the concentrations of MOR and MET with the mean square error values of the validation values were 0.00772 and 0.00892 respectively. In addition, an as-fabricated sensor was applied to detect MOR and MET in the urine samples. MOR and MET detection recovery in urine samples ranged from 95.6 to 105.6. The sensor illustrated an excellent ability to tolerate real sample matrix interferences. The present work proved that CMK-5 mesoporous carbon is a suitable candidate for the construction of sensors. Besides, multivariate calibration methods allow simultaneous detection of multiple analytes with reasonable precision for use in complex matrices.

## Supplementary Information

Supplementary Information.

## Data Availability

The dataset analyzed for the current study is available from the authors on reasonable request.
