# Supplementary material for: Simultaneous electrochemical determination of morphine and methadone by using CMK-5 mesoporous carbon and multivariate calibration
Source: Sci Rep. 2022 May 18;12:8270. doi: 10.1038/s41598-022-12506-9 (PMC9117690; doi:10.1038/s41598-022-12506-9)
Supplement: Supplementary file 1 — Supplementary Information. [file 41598_2022_12506_MOESM1_ESM.docx]

**Simultaneous electrochemical determination of morphine and methadone by using CMK-5 mesoporous carbon and multivariate calibration**

Mohammad Mehdi Habibi ^a^, Jahan B. Ghasemi ^a,^*, Alireza Badiei ^a^ , Parviz Norouzi ^b^

**^a^ School of Chemistry, College of Science, University of Tehran, Tehran, Iran**

**^b^ Center of Excellence in Electrochemistry, Department of Chemistry, University of Tehran**

*Corresponding author: Jahan B. Ghasemi, Email: [jahan.ghasemi@ut.ac.ir](mailto:jahan.ghasemi@ut.ac.ir)


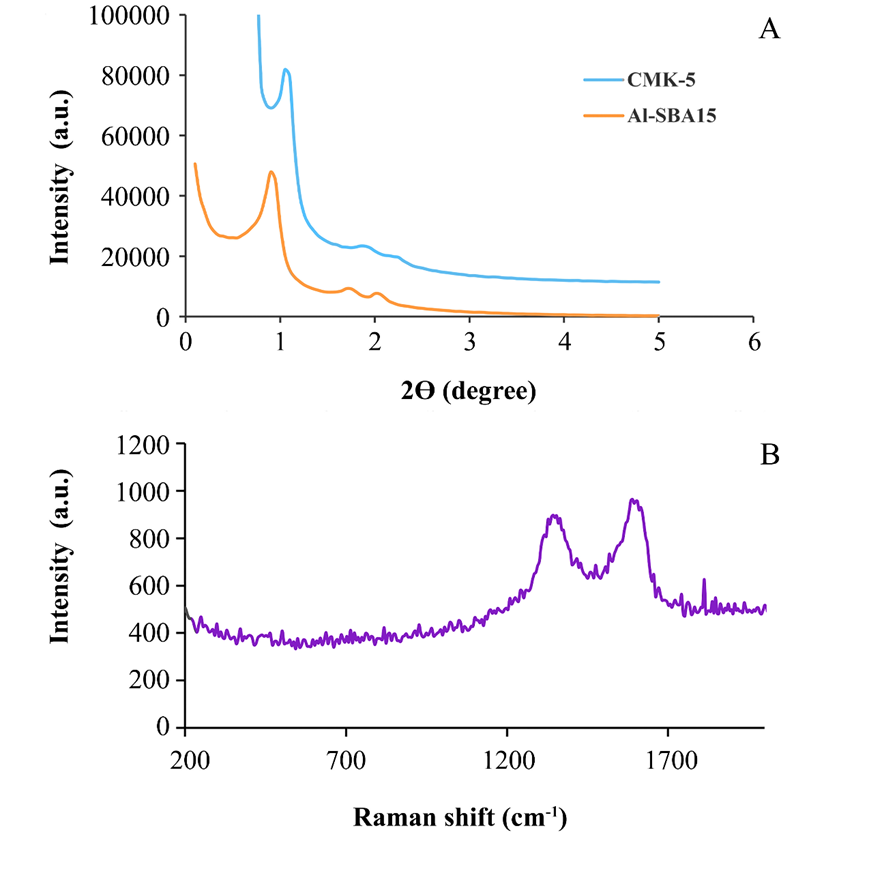


**Fig. S1** A) The XRD pattern of CMK-5 and A1SBA15, and B) the Raman spectra of CMK-5


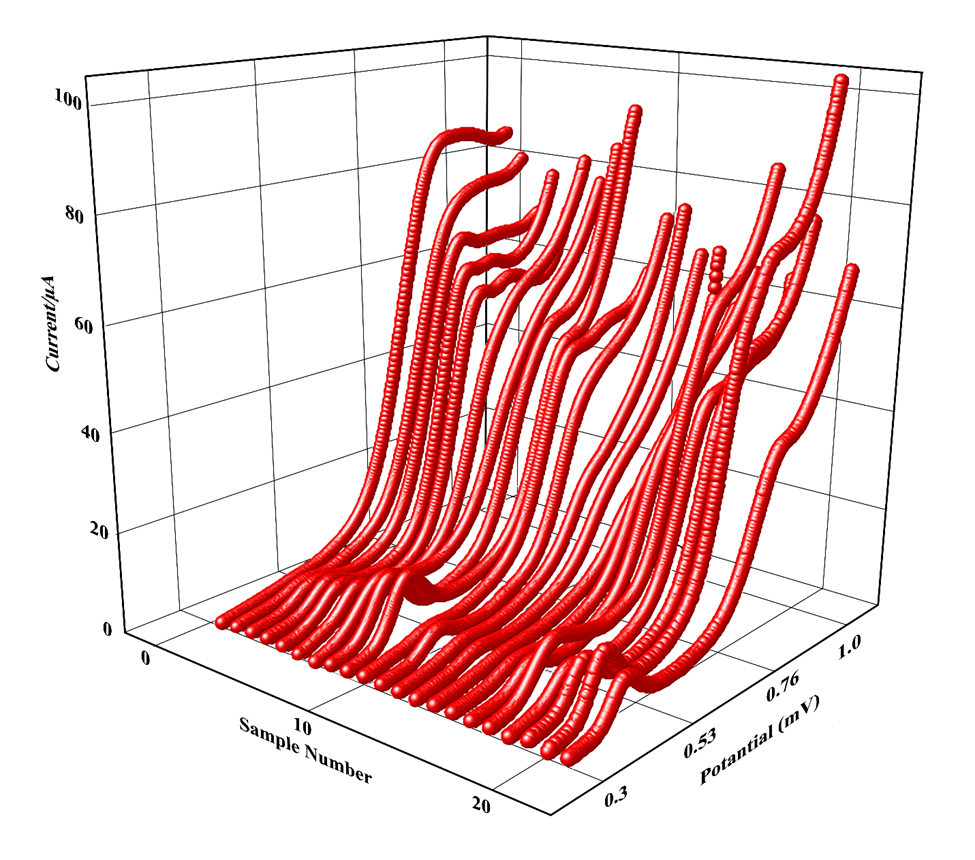


**Fig. S2.** FFT-SWV voltammogram of different concentrations ((Table 1) of MOR and MET mixture solution at FFT-SWV frequency of 256 Hz and the amplitude of 15mV

Fig. S3 FFT-SWV Voltammogram of a mixture of MOR and MET in PB solution with pH = 8.5 with 10 replicates to test the reusability of the sensor. The FFT-SWV frequency of 256 Hz and the amplitude of 15mV

Fig. S4 FFT-SWV Voltammogram of a mixture of MOR and MET in a real urine sample.

**Table. S1**. Train set and a test set of MOR and MET

| Sample number Train | | |
| --- | --- | --- |
| MOR (µM) MET (µM) | | |
| 1  2  3  4 | 0.1  0.5  1  1.5 | 4  3.5  3  2.5 |
| 5  6  7  8  9  10  11  12  13  14 | 2  2.5  3  3.5  4  0.5  1  0.5  0.5  0.2 | 2  1.5  1  0.5  0.1  3  1  0.5  0.2  0.1 |
| Test | | |
| 15  16  17  18  19  20  21 | 0.3  0.2  3.5  1  2.5  4  2 | 0.5  0.3  2  2  3  3  0 |

**Table. S2**. Interference concentration tolerance on determination of MOR and MET

| Species | MOR | MET |
| --- | --- | --- |
| Na^+2^, K^+^, Al^+3^  Cu^+2^, Cr^+2^, Pb^+2^, SO_4_^-2^  Glucose, sucrose, fructose  Ibuprofen  Amphetamine  Ascorbic acid  Codeine | >1000  500  450  300  250  100  50 | >1000  500  450  300  250  150  100 |

**Table. S3**. Recovery studies by PLS multivariable analysis of three urine samples in the presence of MOR and MET

|  |  | Real sample | | | |
| --- | --- | --- | --- | --- | --- |
| Sample number | component | Added (µM) | Found (µM) | Recovery (%) | RSD (%) |
| 1 | MOR | 0.6 | 0.621 | 103.5 | 3.5 |
|  | MET | 0.1 | 0.097 | 97.0 | 2.7 |
| 2 | MOR | 0.1 | 0.105 | 105.0 | 3.9 |
|  | MET | 0.6 | 0.59 | 98.3 | 3.1 |
| 3 | MOR | 0.3 | 0.287 | 95.6 | 2.4 |
|  |  |  |  |  |  |
|  | MET | 0.3 | 0.318 | 105.6 | 2.6 |
